# High Throughput FISH Analysis: A New, Sensitive Option For Evaluation of Hematological Malignancies

**DOI:** 10.4274/Tjh.2012.0033

**Published:** 2013-06-05

**Authors:** Hakan Savlı, Seda Eren, Nilüfer Üzülmez, Zeynep İlkay, Duygu Yavuz, Deniz Sünnetçi, Abdullah Hacıhanifioğlu, Naci Çine

**Affiliations:** 1 Kocaeli University Faculty of Medicine, Department of Medical Genetics, Kocaeli, Turkey; 2 Kocaeli University Faculty of Medicine, Department of Hematology, Kocaeli, Turkey

**Keywords:** Hematologic malignancies, Leukemia, molecular genetics, Microarray

## Abstract

**Objective:** The aim of this study was to determine the efficiency of the high throughput FISH analysis (HTFA) method for detecting genetic alterations in hematological malignancies, which is a new bacterial artificial chromosome array-based approach.

**Materials and Methods:** We performed a HTFA study of bone marrow aspiration and peripheral blood samples of 77 cases (n=19 myelodysplastic syndrome, n=17 acute lymphoblastic leukemia, n=9 chronic myeloid leukemia, n=32 acute myeloid leukemia) with hematological malignancies during the periods of initial diagnosis, treatment, and/or follow-up.

**Results:** Both numerical and structural abnormalities were detected by HTFA. We observed aberrations in 88% of our acute lymphoblastic leukemia patients, 25% of acute myeloid leukemia patients, and 31% of myelodysplastic syndrome patients. In chronic myeloid leukemia cases, aberration was not detected by HTFA.

**Conclusion:** Our results showed that HTFA, combined with other methods, will gradually take a place in the routine diagnosis of hematologic malignancies.

## Abstract (tr)

**Amaç:** Çalışmamızın amacı, hematolojik malignitelerde gözlenen genetik değişimlerin tespitinde, yeni bir BAC tabanlı array yaklaşımı olan HTFA metodunun etkinliğini gözlemlemektir.

**Gereç ve Yöntemler:** Tanı, tedavi ve /veya takip sürecinde olan toplam 77 (n=19 MDS, n=17 ALL, n=9 KML, n=32 AML) hematolojik maligniteli olgudan alınan kemik iliği aspirasyon ve periferik kan materyallerine HTFA metodu uygulandı.

**Bulgular:** Olgularımızda hem sayısal hem de yapısal kromozomal anomaliler tespit edildi. Anomali görülme oranı tanıya göre farklılık göstermektedir. ALL olgularının %88’inde, AML olgularının %25’inde ve MDS olgularının ise %31’inde aberasyon gözlenmiş olup, KML olgularında ise aberasyon saptanmamıştır.

**Sonuç:** HTFA yönteminin, diğer yöntemlerle birlikte kullanıldığında, hematolojik malignitelerin rutin tanı şemalarında yer alabileceğini göstermektedir.

## INTRODUCTION

Hematologic malignancies are characterized by the clonal proliferation and accumulation of malignant immature or mature blood cells in the bone marrow and peripheral blood [1].

Acquired chromosomal abnormalities have been frequently reported in the blood cells of patients with hematological malignancies. Numerical and structural chromosomal abnormalities play an essential role in the pathogenesis of the disease. They also define patient sub- groups and have important prognostic implications [2].

Translocations, deletions, inversions, and other rearrangements are structural chromosomal abnormalities that comprise genes with oncogenic potential, which activate specific differentiation or proliferation pathways and cause the progression of leukemogenesis [1].

Numerical chromosomal changes leading to clones with euploidy or aneuploidy are classified into groups according to chromosome number. Conventional cytogenetics has a very powerful ability to scan the genome for aberrations that involve both gains and losses of portions of the genome, as well as rearrangements within and among chromosomes. Cytogenetic analysis plays an important role in the detection of chromosomal abnormalities in hematologic malignancies [3].

However, cytogenetic analysis has limitations such as low mitotic index, low chromosome band resolution, poor banding quality, and condensed or fuzzy appearance of the chromosomes. In order to overcome these limitations, molecular genetic methods such as fluorescence in situ hybridization (FISH), quantitative real-time polymerase chain reaction (Q-RT-PCR), and array comparative genomic hybridization (CGH) have emerged for diagnosis of hematologic malignancies [4,5].

CGH is a method for determining copy number gains and losses between 2 samples of DNA by competitively hybridizing differentially labeled DNA to metaphase chromosomes. This technology was first developed as a research tool for the investigation of genomic alterations in cancer [6,7]. DNA copy number alterations associated with chromosome abnormalities can be analyzed with high resolution by array CGH. Array CGH has been a valuable tool in the analysis of hematological malignancies that provides knowledge of the genomic alterations in this heterogeneous group of diseases [8,9,10].

A new technology called high throughput FISH analysis (HTFA) is different from conventional cytogenetics techniques and able to detect more than 1 deletion- duplication region at the same time with high resolution at 300-500 kb. It is a bacterial artificial chromosome (BAC) array platform introduced for use in 2010. HTFA panels contain 31 regions on somatic chromosomes that are related to hematologic malignancies. HTFA might be thought of as a kind of array CGH technology, but it is not accepted as a traditional glass microarray platform due to the fact that multi-FISH probes are designed with BAC.

HTFA can even further magnify the precision of diagnosis and prognosis, as well as provide a single standardized platform [11]. Moreover, DNA copy number changes throughout the whole genome can be obtained from very small amounts of DNA. A major limitation of this array CGH-based technique is that balanced chromosomal rearrangements, such as reciprocal translocations or inversions, cannot be detected [12]. This requires additional cytogenetics and molecular cytogenetic approaches (e.g., FISH, karyotyping analysis, and Q-RT-PCR) to reveal cytogenetic changes. One other disadvantage of HTFA is that commercial platforms have still been insufficient for detecting mosaicism, while cut-off values differ among laboratories [9,12,13].

We applied HTFA analyses to 77 leukemia patients to determine unbalanced structural abnormalities and numerical abnormalities in 2010 and 2011 in the Medical Genetics Department of Kocaeli University. Our findings represent one of the first observations related to HTFA of hematological malignancies.

## MATERIALS AND METHODS

We performed HTFA study on the bone marrow aspiration and peripheral blood samples of 77 patients (n=19 myelodysplastic syndrome [MDS], n=17 acute lymphoblastic leukemia [ALL], n=9 chronic myeloid leukemia [CML], n=32 acute myeloid leukemia [AML]) with hematological malignancies during the periods of initial diagnosis, treatment, and/or follow-up by the Hematology Department of Kocaeli University. Age, sex, white blood cell count, hemoglobin, diagnosis, and Q-RT-PCR findings of patients with abnormal HTFA results are listed in the Table 1.

Genomic DNA was obtained by using Magna Pure Compact Nucleic Acid Isolation Kit I (Roche Diagnostics GmbH, Germany). The 260 and 280 nm wavelength absorbances were measured by spectrophotometer (ND- 1000, NanoDrop, USA). gDNAs with a A260/A280 ratio between 1.7 and 2.0 were used. Reference and samples were labeled with Cy5-dCTP and Cy3-dCTP using a fluorescent labeling system kit (dCTP/BAC, BlueGnome Ltd., UK). After incubation of 16 to 20 h at 37 °C, samples were purified with AutoSeq G50 columns (BlueGnome Ltd.). Human Cot-I DNA (BlueGnome Ltd.) was added to avoid consecutive matches from repetitive regions. A hybridization mixture was added and denaturated at 75 °C. Samples were loaded on HTFA platforms (BlueGnome Ltd.) and hybridized at 47 °C for 16-21 h. Finally, the HTFA platform was scanned (Agilent Microarray Scanner, Agilent Technologies, USA) and analyzed with BlueFuse Multi v2.1 software (BlueGnome Ltd.). 

## RESULTS

Aberrations were detected in 29 of 77 patients. We identified numerical and structural abnormalities in 8 AML, 15 ALL, and 6 MDS patients. All numerical and structural aberrations detected by HTFA and Q-RT-PCR are listed in the Table.

CML patients (n=9) did not show any aberration with HTFA. More than 1 aberration existed in 21 of 29 cases. Detected recurrent numerical aberrations were as follows: trisomy 8 in 1 ALL and 4 AML patients; trisomy 21 in 1 AML, 2 MDS, and 4 ALL patients; loss of chromosome Y in 1 AML and 2 ALL patients; loss of chromosome X in 2 MDS patients; gain of X in 3 ALL patients; and trisomy 10 in 2 ALL and 1 AML patients. Genome views of patients with normal karyotype and several abnormalities are shown in Figure 1A, B, C, D.

## DISCUSSION

Detecting genetic aberrations in hematological malignancies provides essential information for diagnosis and prognosis to clinicians. At present, conventional cytogenetics methods such as G or R banding are the most widely used methods for identifying chromosome aberrations in cancer cells. These techniques have always been limited by the complexity and poor morphology of cancer chromosomes or the requirements of fresh material (dividing cells) to obtain metaphases. To overcome these problems, Q-RT-PCR, FISH, and high throughput (genomic) approaches such as array CGH are being used [6,9,13].

In this study, we aimed to examine the HTFA method’s effi ciency for detecting genetic aberrations, which are important for the diagnosis and prognosis of leukemia.

Of our patient group, 62% had normal HTFA results and 38% had numerical and structural chromosomal abnormalities. The incidence of chromosomal abnormalities in hematologic malignancies changed according to the type of disease. We identifi ed chromosomal abnormalities in 25% of AML and 31% of MDS cases. In ALL patients, the anomaly rate (88%) was higher than the anomaly rate (68%) detected by conventional cytogenetics in similar studies [14]. The major reason for differences between incidences is that HTFA enables the observation of submicroscopic changes that cannot be detected by conventional cytogenetics. Additionally, this method does not require the obtaining of metaphases, and thus aberrations can be determined with all samples.

We observed both chromosome losses and gains as numerical chromosomal abnormalities related to leukemia. Trisomy 8 is the most common single chromosomal abnormality in AML and MDS, representing approximately 6%-11% of these myeloid leukemias. It also has been reported in patients with lymphoid leukemias. We detected this anomaly in 4 of 37 (11%) AML and 1 of 17 (5%) ALL patients. However, although trisomy 8 is the most common numerical abnormality, the prognostic impact of the anomaly is not well known. As seen in recent studies, it does not confer a particularly favorable prognosis for AML [15,16].

Another numerical chromosomal abnormality of our study was trisomy 21. It was detected in 1 AML, 4 ALL, and 2 MDS cases. Acquired trisomy 21 is the second most common trisomy in MDS and AML after trisomy 8. Nevertheless, the clinical and prognostic impacts of this anomaly in myeloid leukemias remain incompletely characterized. One AML patient (Case 10H31) had constitutional trisomy 21. Clinical observations showed that there is a 10 to 20 times increased risk of leukemia in individuals with constitutional trisomy 21. Trisomy may lead to leukemogenesis, whereby the presence of an increased copy number of certain genes gives a cell survival advantage and hence neoplastic potential. As an acquired anomaly, it is thought to lead to poor prognosis in MDS [17].

Recurrent chromosomal deletions and duplications are observed in leukemia patients as unbalanced structural chromosomal abnormalities. Chromosomal deletions were found more frequently than duplications in our samples, consistent with the literature [13]. We detected 24 different microscopic (>5 Mb) and submicroscopic deletions (<5 Mb) in our study. Deletions in 4 patients (18%) were submicroscopic deletions, which cannot be visualized by conventional cytogenetics. One of the submicroscopic deletions that we observed was del(13)(q13.3q21.32). Generally, the 13q anomaly was found at diagnosis, while, in rare cases, the anomaly appeared during the course of the disease. The deletion has been described as interstitial in most cases, with the following breakpoints: q13-q21 (most frequently), q13-q22, q14-q22, and q12-q21. Loss of material at band 13q14-21 is common to all cases. The data indicate the presence of 2 distinct breakpoint cluster regions: centromeric of RB1 in myeloid malignancies and distal to RB1 in some lymphoid B-cell and T-cell malignancies. Our deletion region covered the q14q21 critical region, which includes the RB1 gene. The RB1 gene is a tumor suppressor gene whose function is closely related to cell-cycle control. The 13q14 deletions usually do not lead to inactivation of the RB1 gene, and del(13q) is associated with the presence of one intact RB1 gene on the homologous chromosome, implying the role of an adjacent locus that may harbor important tumor suppressor gene(s) [18].

It is expected to detect aberrations affecting expression of immunoglobulin chain genes on 14q32 or T-cell antigen receptor genes on 7q34 in leukemia cases. We detected del(14)(q32.33) as a submicroscopic deletion in one AML patient. This region includes the D9 immunoglobulin heavy chain variable region. The T-cell antigen receptor B mRNA region is located on 7q34. del(7)(q21.3q36.3) was detected in one patient with AML and the aberration includes the 7q34 region, which is very variable [13].

del(5)(q23.3q35.3) and del(5)(q13.2q35.3) were seen in 1 AML and 1 MDS patient. Deletion of 5q can be observed in both de novo and therapy-related AML. It is also seen as monosomy 5. On the other hand, it is one of the most common structural rearrangements in MDS (10%), seen as an isolated abnormality or with additional karyotypic anomalies. It is also observed in AML, with important prognostic signifi cance. Prognosis of AML with 5q-/-5 is generally unfavorable, associated with rapid disease progression and poor outcome and survival, especially when it is seen as a part of complex karyotypes. The most commonly observed interstitial deletions are del(5)(q13q31), del(5)(q13q33), and del(5)(q22q33), forming a commonly deleted region at 5q31-q32 [19,20].

We detected 9p abnormalities in 2 patients with ALL and AML. In the ALL patient, del(9p) abnormality was seen, and del(9)(p21p13.2) was seen in a childhood AML patient. Deletions of the 9p21 chromosomal region are frequent in childhood ALL and encompass CDKN2A (MTS1), a gene encoding both p16INK4a and p14ARF. p16INK4a, an inhibitor of cyclin-dependent kinase, inhibits Rb phosphorylation, whereas p14ARF activates TP53 via interaction with the MDM2 protein 1. Hypermethylation of the promoter has been shown to be an alternate way of inactivation for these proteins in a variety of malignancies, including ALL [21].

All gains in the genome, such as duplications, triplications, or amplifi cations, were evaluated as duplications by HTFA. We detected duplicated chromosome regions in 12 patients and 4 of them had more than 1 duplication.

Marker chromosomes whose origin cannot be identifi ed by G banding are seen in hematological malignancies. Marker chromosomes include duplicated regions of various chromosomes that cannot be identifi ed by G banding. Additional molecular methods are required to determine genes that are present in marker chromosomes [5]. A considerable advantage of HTFA is that this method is able to identify and determine all duplicated regions and genes that exist on marker chromosomes. The determining of duplicated genes provides valuable information for prognosis and diagnosis. Four patients had more than 1 duplication. These patients may have extrachromosomal/ intrachromosomal duplications or amplifi cations that form marker chromosomes.

The c-myc gene is a proto-oncogene that is located on 8q24. Rearrangements and amplifi cations of myc are seen in leukemia. It plays a role in cellular processes such as cell cycle progression, apoptosis, metabolism, immortality, and adhesion [22]. Duplications including the 8q24 region were observed in 1 ALL, 1 AML, and 1 MDS patient.

Translocations having diagnostic and prognostic value for leukemia should be identifi ed by appropriate methods. t(9;22) is the most common translocation in CML that can be detected by conventional cytogenetics, FISH, and Q-RTPCR methods. Additionally, it can be seen in ALL or AML as a poor diagnosis indicator [23]. We observed t(9;22) in 1 AML patient with Q-RT-PCR. In the HTFA results, there were no alterations in the breakpoints of chromosomes 9 and 22. This fi nding indicates that HTFA is not able to detect balanced chromosomal rearrangements such as translocations. The inability to detect balanced translocations is the main limitation of this method for leukemias.

Using conventional cytogenetic techniques, many karyotypic changes have been detected in metaphase preparations, which proved to be important diagnostic and prognostic markers for hematological malignancies. Conventional cytogenetic methods have some limitations such as low mitotic index and poor morphology of cancer chromosomes [8]. No information about genetic alterations can be observed because of unsuccessful cell culturing. On the other hand, some submicroscopic alterations cannot be visualized because of poor chromosome morphology and lower resolution of G banding. These limitations lead to the assessment of genetically altered cells as normal cells. The FISH method was developed to overcome these diffi culties with anomaly-specifi c probes. Based on karyotypic information, highly specifi c probes have been designed for karyotype analyses of interphase nuclei. It is very important to clarify that FISH is not a technique designed to look for new aberrations; it only detects alterations of the tested probe and the status of the rest of the genome remains hidden [3,9,12].

As a conclusion HTFA is a BAC-based multi-FISH analysis technique designed to overcome the limitations of FISH and conventional cytogenetics. High-resolution full-genome screening with HTFA will help to detect the candidate genes in leukemia and acquire more information about the mechanisms of leukemogenesis. To achieve further information about the effectiveness of HTFA, confi rmative methods and studies with large numbers of patients are required. Although it has several limitations, we think that HTFA, combined with other methods, will gradually take its place in the routine diagnosis of hematologic malignancies.

Conflict of Interest Statement

The authors of this paper have no confl icts of interest, including specific financial interests, relationships, and/ or affi liations relevant to the subject matter or materials included.

## Figures and Tables

**Table 1:**
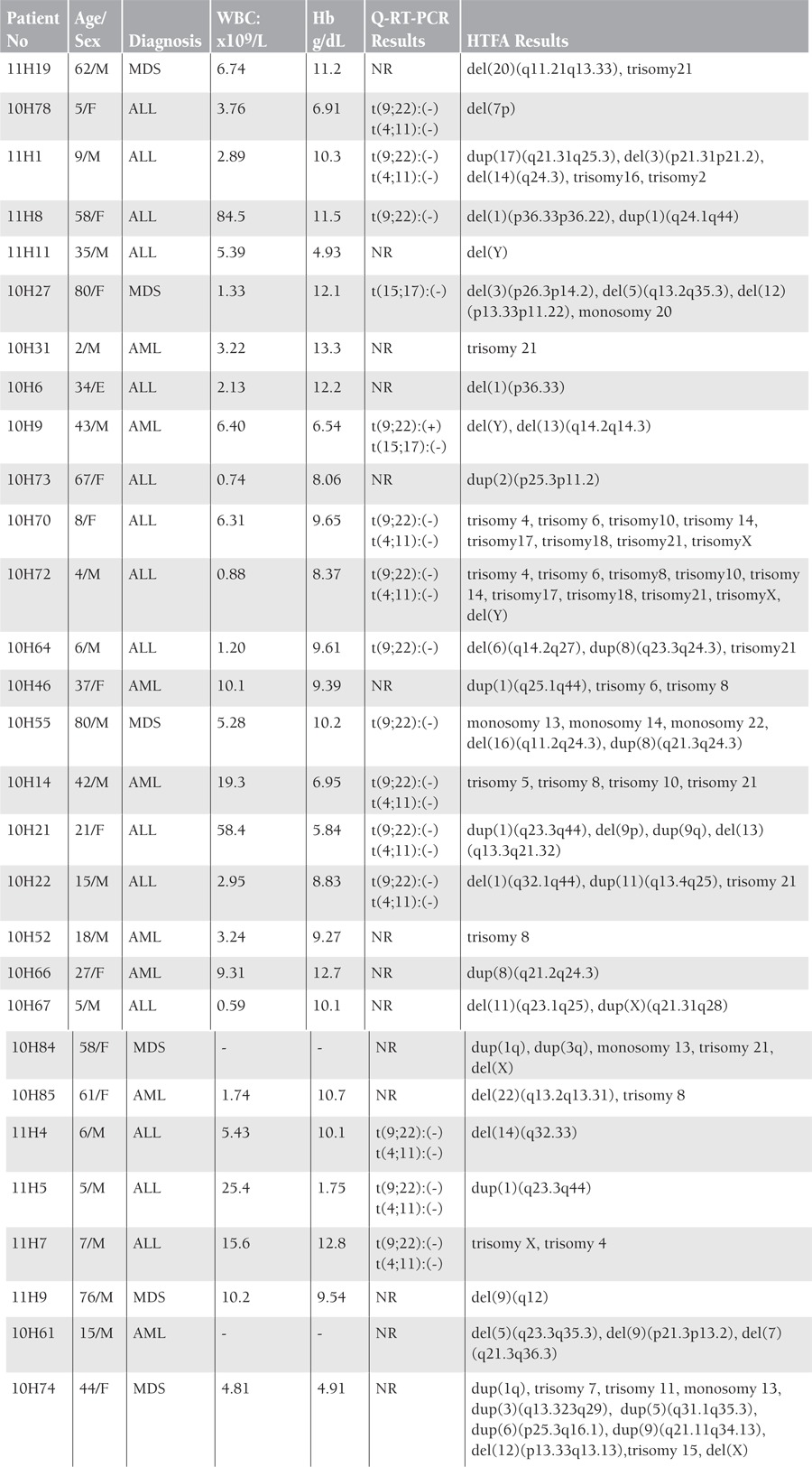
Age, sex,WBC, diagnosis, Hgb, Q-RT-PCR and abnormal HTFA results of patients are listed. Abbreviations: NR, not requested by clinician.

**Figure 1A:**
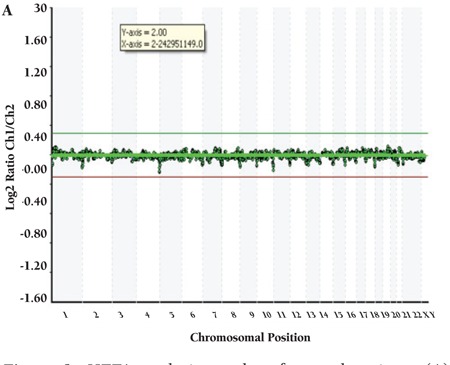
HTFA analysis results of several patients (A) patient with normal karyotype.

**Figure 1B:**
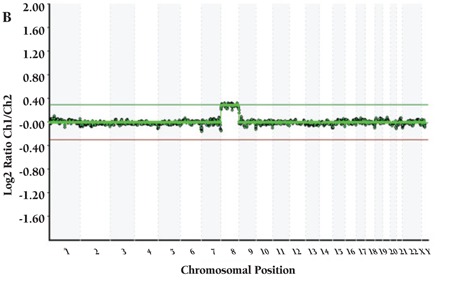
(B) Patient with trisomy 8.

**Figure 1C:**
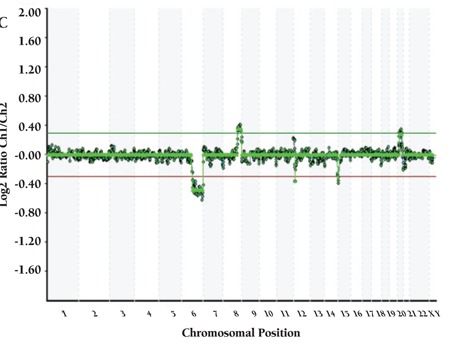
(C) Patient with del(6)(q14.2q27), dup(8)(q23.3q24.3),trisomy 21.

**Figure 1D:**
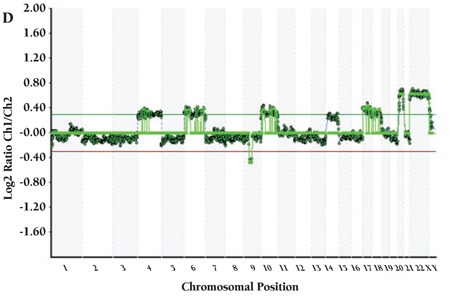
(D) Patient with trisomies of chromosomes 4, 6, 8, 10,14,17, 18, 21, X and del(Y).
